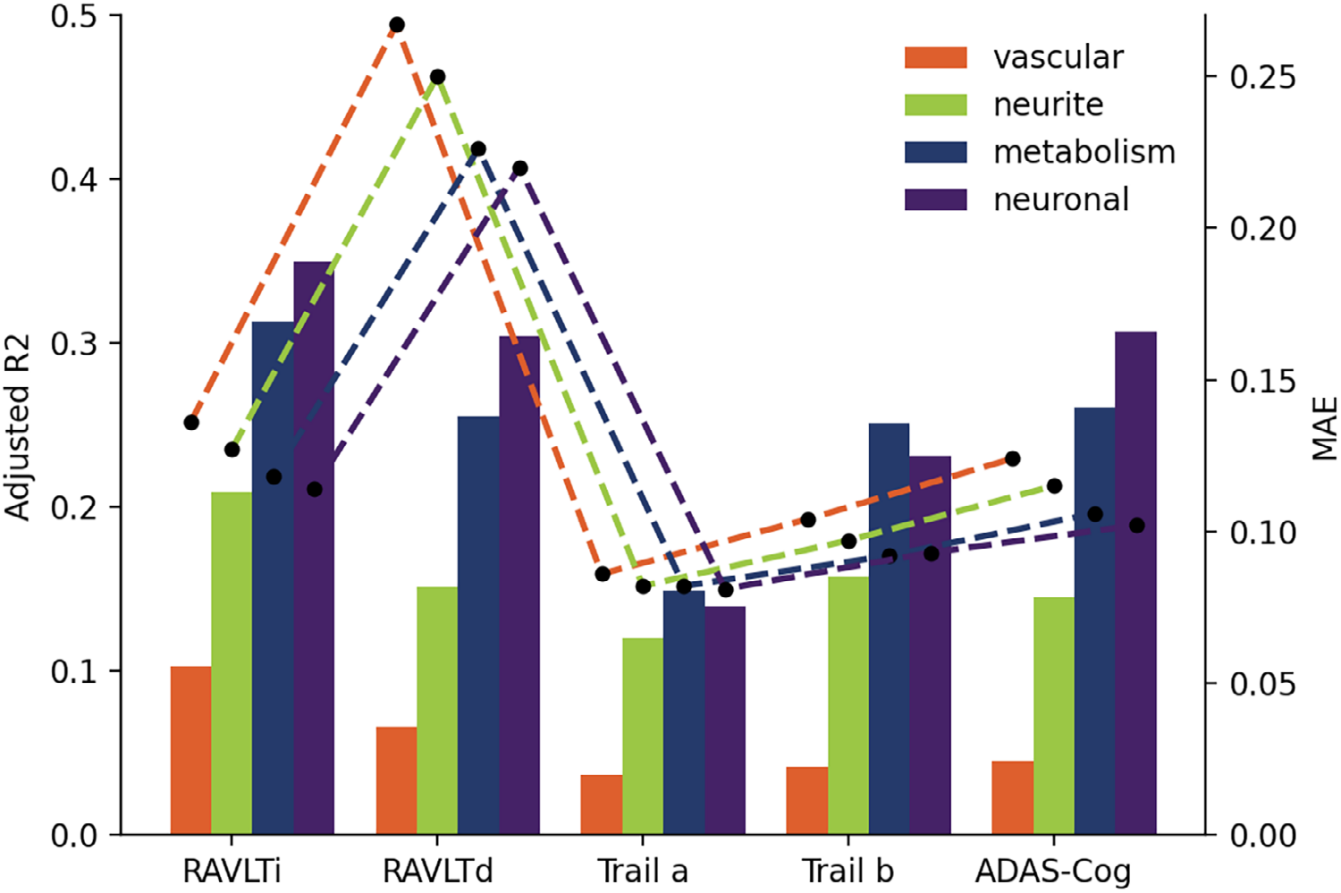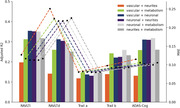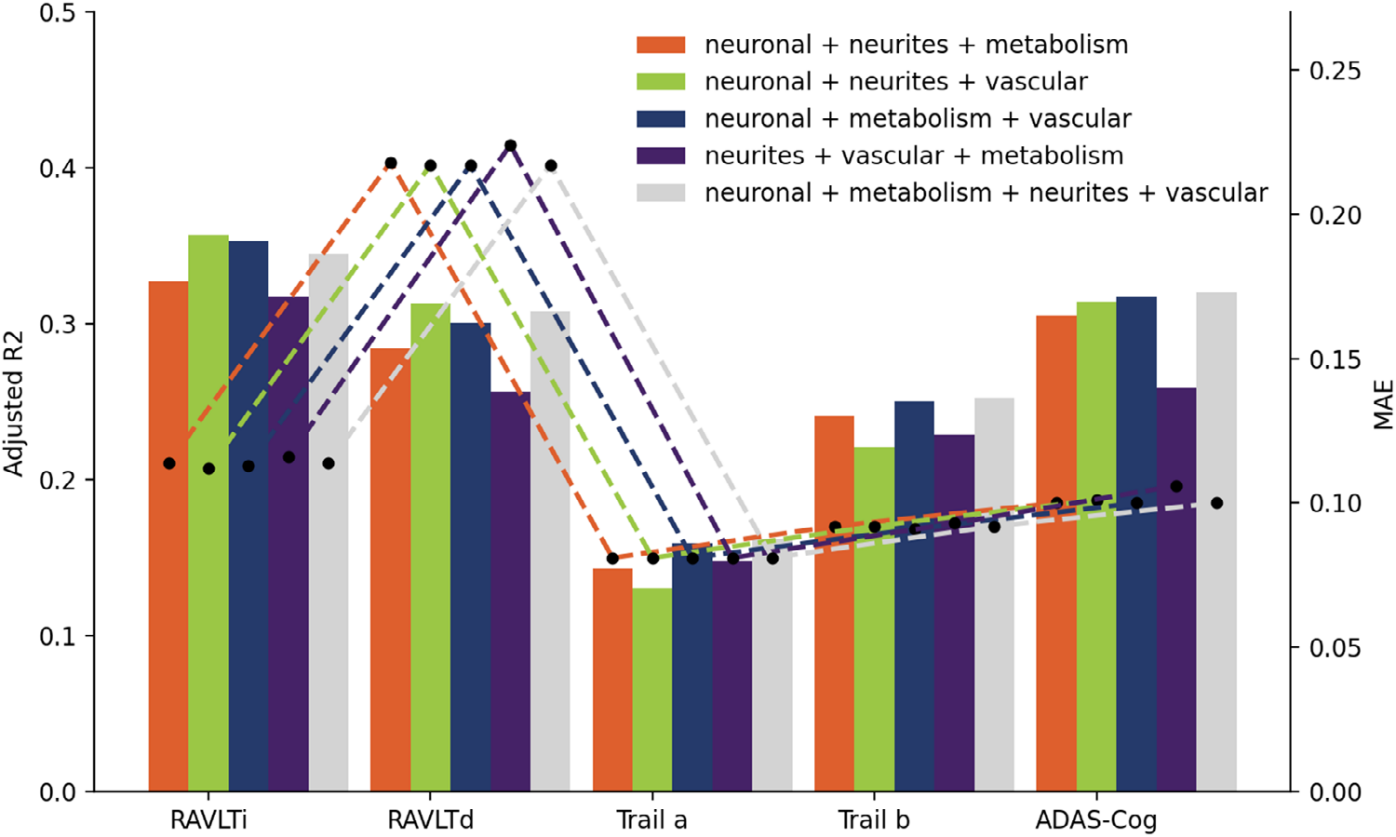# Predicting cognitive function from multimodal mesoscale neuroimaging data

**DOI:** 10.1002/alz.086415

**Published:** 2025-01-09

**Authors:** Simon Duchesne, Olivier Potvin, Louis Dieumegarde

**Affiliations:** ^1^ Centre de recherche de l’Institut Universitaire de Cardiologie et de Pneumologie de Québec, Quebec, QC Canada; ^2^ Faculty of Medicine, University Laval, Quebec City, QC Canada

## Abstract

**Background:**

Our objective was to assess individual and joint relationships between various mesoscale indicators of brain health (e.g., neuronal, metabolic, and vascular integrity) and cognitive function.

**Method:**

We used 7224 timepoint measures originating from 1825 participants of the ADNI study who were cognitively healthy or with mild cognitive impairment at initial assessment. Cognitive function outcomes were test results on the Alzheimer’s Disease Assessment Scale–Cognitive Subscale (ADAS‐Cog), the Rey auditory Verbal Learning Test immediate recall (RAVLTi), the Rey auditory Verbal Learning Test delayed recall (RAVLTd), the Trail Making Test A (Trail a), and the Trail Making Test B (Trail b) tasks. Four families of markers were used. First were three families of mesoscale neuroimaging assessed by MRI for 88 brain regions: 1. Neuronal integrity was estimated by FreeSurfer 6.0 produced volumes extracted from T1‐weighted (T1w) images; 2. Neurite integrity was estimated through T1w/T2‐weighted MRI ratios; 3. Cerebral metabolism integrity was measured by synthetic fluorodeoxyglucose‐positron emission tomography, generated by a model based on a latent‐space regularized generative adversarial net from T1w MRIs. Secondly, 4. Systemic markers of vascular health‐related factors were included in the model (history of cardiovascular diseases, alcohol abuse, smoking history, systolic and diastolic blood pressure, history of hypertension, type 2 diabetes, Hachinski score).

Cross‐sectional and future cognitive outcomes were all predicted in a single machine learning model using all combinations of predictor families at baseline, time interval between outcomes and baseline as long as sex and education. Three types of regression models were tested (Ridge, Partial Least Squares and Support Vector).

**Result:**

Individually, neuronal and metabolism integrity were the best predictors of cognitive function (Figure 1; R^2^: .15‐.35 and MAE in Z score: .08‐.23). The combination of two or more mesoscale brain health markers improved predictions, with the best combination being neuronal integrity with metabolism (Figures 2‐3; mean R^2^: .28; mean MAE: .12). However, increasing the number of markers led to similar results.

**Conclusion:**

While the prediction of cognition improved with a combination of brain health mesoscale markers, a plateau seems to be reached after combining two families.